# Development of Heterogeneous Olympic Medal Metal Nanoparticle Catalysts for Environmentally Benign Molecular Transformations Based on the Surface Properties of Hydrotalcite

**DOI:** 10.3390/molecules15128988

**Published:** 2010-12-08

**Authors:** Kiyotomi Kaneda, Takato Mitsudome, Tomoo Mizugaki, Koichiro Jitsukawa

**Affiliations:** 1 Department of Materials Engineering Science, Graduate School of Engineering Science, Osaka University, 1-3, Machikaneyama, Toyonaka, Osaka 560-8531, Japan; 2 Research Center for Solar Energy Chemistry Osaka University, 1-3, Machikaneyama, Toyonaka, Osaka 560-8531, Japan

**Keywords:** heterogeneous catalyst, Olympic medal metal, nanoparticles, hydrotalcite

## Abstract

In this review, we describe the development by our research group of highly functionalized heterogeneous Olympic medal metal (gold, silver, and copper) nanoparticle catalysts using hydrotalcite as a support, aimed towards Green and Sustainable Chemistry. Olympic medal metal nanoparticles can cooperate with the basic sites on the hydrotalcite surface, providing unique and high performance catalysis in environmentally-benign organic transformations such as aerobic oxidation of alcohols, lactonization of diols and selective deoxygenation of epoxides and nitro aromatic compounds.

## 1. Introduction 

In designing heterogeneous catalysts, the creation of cooperative action between active metal species and an inorganic material support is a promising methodology for providing unique and highly active catalysis. Inorganic crystallites surrounding the active metal species can be considered as macro ligands that lead to greatly altered catalytic performance, while organic ligands can be tuned to improve the catalytic activity of the metal center for the target reactions.

Hydrotalcite (HT, Mg_6_Al_2_(OH)_16_CO_3_•nH_2_O) is a layered anionic clay consisting of a positively charged two-dimensional brucite layer with anionic species such as hydroxide and carbonate located in the interlayer. HT has attracted attention not only for adsorption and drug delivery applications, but also for catalysis due to the following characteristics: (i) the cation-exchange ability of the brucite layer; (ii) the anion-exchange ability of the interlayer; (iii) the tunable basicity of the surface; and (iv) the adsorption capacity. Herein, we describe our recent research on the unique catalytic performance of hydrotalcite-supported Olympic medal metal (gold, silver, and copper) nanoparticles for various organic transformations. The concerted effect between the metal nanoparticles and the base sites of hydrotalcite leads to high performance heterogeneous catalysts.

## 2. Oxidation of Alcohols

### 2.1. Aerobic Oxidation of Alcohols Using Au/HT

The oxidation of alcohols is currently a significant issue in many important fields [1,2,3] such as organic synthesis, hydrogen storage/release and transformation of renewable biomass resources. For this purpose, various oxidizing reagents such as permanganate and dichromate have long been used; [4,5,6], however these reagents are expensive and/or toxic and result in the production of large amounts of waste. Therefore, much attention has been directed toward the development of reusable solid catalysts that employ abundant molecular oxygen (O_2_) as the primary oxidant and produce water as the sole byproduct [7,8,9,10,11,12,13].

We synthesized uniformly dispersed Au nanoparticles on hydrotalcite (Au/HT) that act as a highly active solid catalysts for oxidation of various alcohols under the following mild reaction conditions: i) no requirement for additives; ii) atmospheric O_2_; and iii) ambient temperature. The Au/HT catalyst was also effective towards less reactive cyclohexanol derivatives, producing the corresponding cyclohexanones in excellent yields. Moreover, the Au/HT catalyst can be reused without any loss of its activity or selectivity [14].

Hydrotalcite-supported gold nanoparticles (Au/HT) were synthesized using a deposition-precipitation method followed by treatment with KBH_4_. Au/HT oxidized a wide range of alcohols to the corresponding carbonyl compounds with high yields under mild reaction conditions (Table 1). Benzylic secondary alcohols were found to be good substrates (entries 1, 5 and 6). Oxidation of benzylic primary alcohols (entries 7 and 8) also proceeded, but with slightly lower yields. An aliphatic secondary alcohol was oxidized quantitatively (entry 9), while the aliphatic primary substrate 1-octanol (entry 10) was not reactive. Various allylic alcohols (entries 11-13) were effectively oxidized to the corresponding α,β-unsaturated carbonyl compounds. Au/HT was also tolerant of nitrogen atoms in a heteroaromatic ring (entry 14). Alicyclic alcohols (entries 15-18), including bulky alcohols, were also highly reactive. Interestingly, cyclohexanol derivatives were effectively converted to cyclohexanones (entry 19-21). Furthermore, the Au/HT catalyst was applicable under neat conditions. Specifically, 1-phenylethanol (30 mmol, 3.7 g) was oxidized in the absence of solvent to give a 93% yield of acetophenone (3.4 g) with 99% selectivity after 24 h. The reaction exhibited a turnover number (TON) and turnover frequency (TOF) (200,000 and 8,300 h^-1^, respectively) comparable to those reported for other catalyst systems [10,11,12,13].

The reusability of the Au/HT catalyst was investigated via a recycling experiment involving oxidation of 1-phenylethanol, where the Au/HT catalyst was filtered and reused under identical conditions in three consecutive runs. As shown in Table 1 (entries 2-4), the recovered Au/HT exhibited consistent catalytic activity. TEM images of the reused Au/HT catalyst showed that the average size and distribution of the Au nanoparticles were not significantly altered and that Au aggregates were not formed. This result is consistent with the retention of catalytic activity of Au/HT in the recycling experiment.

We also investigated the relationship between the size of the Au nanoparticles and the catalytic activity for the above oxidation (Table 2). We found that the particle size of Au nanoparticles could be controlled by selection of the reducing reagents used during preparation of the catalyst; specifically, the size of the gold nanoparticles decreased in order for the following reducing reagents: hydrazine > molecular hydrogen > potassium borohydride. The yields of acetophenone increased with decreasing Au nanoparticle size.

**Scheme 1:**
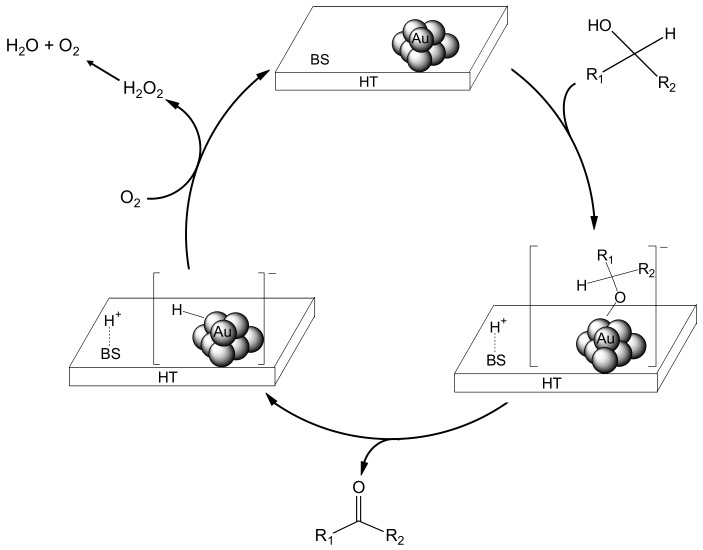
Plausible Mechanism for the Au/HT-catalyzed Aerobic Oxidation of Alcohols Involving Participation of Au NPs and a Basic Site of HT (Represented by BS).

The effect of inorganic supports on the oxidation activity of the Au nanoparticles was also investigated. As shown in Table 2, Au/MgO (entry 4) and Au/Al_2_O_3_ (entry 7) provided moderate yields of acetophenone, whereas Au/TiO_2_ (entry 10) and Au/SiO_2_ (entry 12) were ineffective as catalysts. However, for Au/TiO_2_, the addition of Na_2_CO_3_ as a base to the reaction mixture significantly improved the yield of acetophenone (entry 10 *vs*. 11) [15]. These results indicate that a concerted catalysis occurring between the base sites of the inorganic support and the Au nanoparticles is essential for achieving high catalytic activity in the oxidation of 1-phenylethanol. In the Au/HT-catalyzed aerobic oxidation of alcohols, the base sites of HT may extract a proton from the hydroxyl group in the alcohol, promoting the facile formation of a Au-alcoholate species. The Au-alcoholate species could then undergo β-hydride elimination, yielding the carbonyl product accompanied by the Au-hydride species. The catalytic cycle would be complete after reaction of the Au-hydride species with O_2_ (Scheme 1).

### 2.2. Lactonization of Diols Catalyzed by Au/HT Using O_2_

The selective synthesis of lactones is of considerable interest to both academic and industrial chemists because lactones are ubiquitous among natural and synthetic organic compounds [16]. Among the various methods of synthesizing lactones, oxidative lactonization of α,ω-diols is one of the most promising methodologies for industrially acceptable processes. To date, several homogeneous catalytic lactonizations have appeared utilizing Ru, [17,18,19,20,21,22,23] Rh, [24] Pd [25,26] and Ir [27] as catalysts combined with organic co-oxidants such as ketones and alkenes. We found that Au/HT showed high catalytic activity for lactonization of α,ω-diols under mild reaction conditions [28]. The Au/HT catalyst can overcome problems such as the requirement for high temperatures, additives and high catalyst loadings that have plagued the previously reported lactonization catalyst systems [21,22,23,25].

The Au/HT catalyst oxidizes a wide range of α,ω-diols to afford the corresponding lactones in excellent yields (Table 3). The olefinic group-bearing diol *cis*-2-butene-1,4-diol was chemoselectively converted to the unsaturated lactone 2(5H)-furanone with suppression of hydrogenation of the olefinic group (entries 8 and 9). The catalyst system was also applicable for the synthesis of heterocyclic lactones incorporating oxygen and nitrogen atoms. For example, diethyleneglycol and *N*-methyl-diethanolamine gave the corresponding lactones in high yields (entries 14 and 15). A possible reaction mechanism for the lactonization is proposed based on cooperative action between Au and HT. An alcoholate species could allow smooth oxidation of one of the hydroxyl groups of the diol to the hydroxyaldehyde, which would likely be in equilibrium with the lactol. Subsequent further oxidation of the lactol would furnish the corresponding lactone (Scheme 2).

### 2.3. Dehydrogenation of Alcohols Using Cu/HT

The development of a reusable and less expensive metal catalyst for the efficient dehydrogenation of alcohols is also a major challenge from both ecological and practical points of view. [29,30] We thus developed a highly efficient heterogeneous catalytic system using hydrotalcite-supported Cu nanoparticles (Cu/HT) having a mean diameter of 7.5 nm that can successfully promote the oxidant-free dehydrogenation of various alcohols and diols (Table 4) [31,32].

In this reaction, an equimolar amount of molecular hydrogen and product is formed.

## 3. Selective Deoxygenation of Epoxides and Nitro Aromatic Compounds

### 3.1. Deoxygenation of Epoxides Using Au/HT-Alcohols

**Scheme 3:**
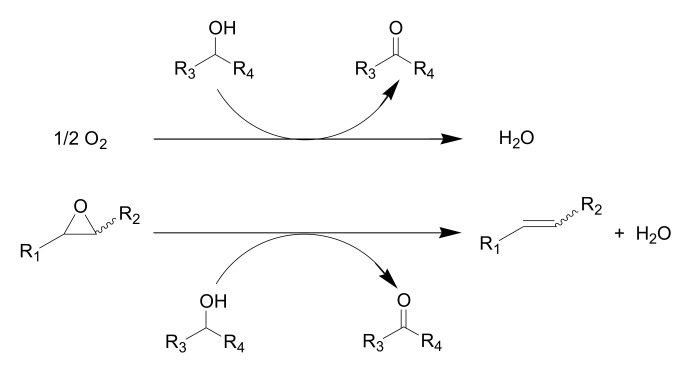
The Oxidation of Alcohols Using O_2_ vs. Deoxygenation of Epoxides Using Alcohols.

In the course of our study of Olympic medal metal nanoparticle catalysis for the aerobic oxidation of alcohols, we envisioned that if epoxides could act as hydrogen accepters in place of molecular oxygen, the metal nanoparticles could act as effective catalysts for deoxygenation of epoxides using alcohols as oxygen acceptors (Scheme 3).

Deoxygenation of epoxides to alkenes is a valuable reaction in organic synthesis because it allows the use of the oxirane ring as a protective group for carbon-carbon double bonds. [33,34,35] The transformation is also of great importance in biological chemistry for the reproduction of vitamin K in the vitamin K cycle [36,37]. In addition, it has recently been applied as a quantification method for determination of the epoxide content in graphite epoxide or oxygenated carbon nanotubes [38,39]. Stoichiometric deoxygenation of epoxides has been carried out using a variety of reagents [40]. These reagents, however, are often toxic and/or are employed in large excess, resulting in the production of undesired waste. Several successful catalyst systems have also appeared to date, but these systems suffer from the need for hazardous reductants, are typically air- and moisture-sensitive and often exhibit low catalytic activities and selectivities [41]. Therefore, the development of an efficient catalytic system for deoxygenation of epoxides remains of great importance.

We discovered the intrinsic ability of gold and silver nanoparticles to catalyze de-epoxidation; gold and silver nanoparticles supported on hydrotalcite (Au/HT and Ag/HT) can promote highly efficient catalytic deoxygenation of epoxides into alkenes using alcohols [42]. To the best of our knowledge, this is the first report of the catalytic deoxygenation of epoxides using gold and silver nanoparticles.

**Scheme 4:**
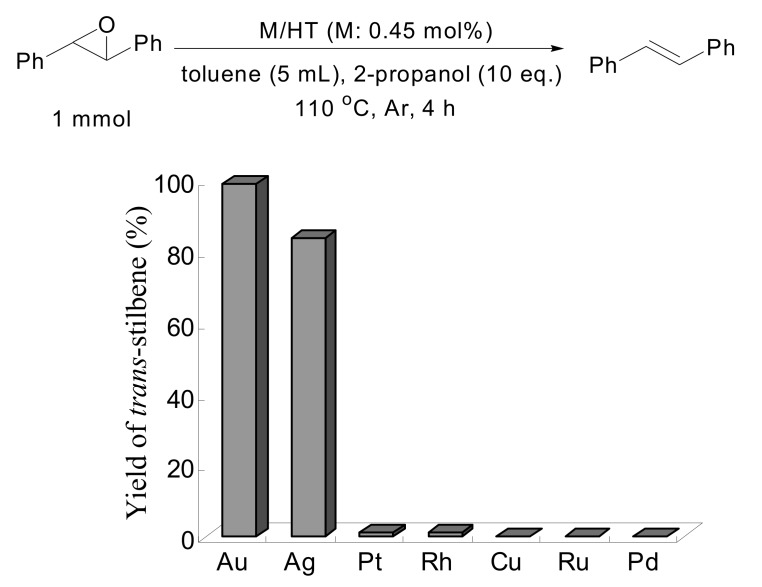
Deoxygenation of *trans*-Stilbene Oxide Using Hydrotalcite-supported Metal Particles.

A mixture of solid Au/HT, *trans*-stilbene oxide and 2-propanol in toluene was stirred at 110 °C under an Ar atmosphere for 4 h. Selective deoxygenation of *trans*-stilbene oxide occurred to afford *trans*-stilbene in 99% yield without any byproducts such as 1,2-diphenylethane, 1,2-diphenylethanol or benzyl phenyl ketone, with the reaction occurring through hydrogenation and/or isomerization of *trans*-stilbene oxide. Other metal nanoparticles on HT having catalytic potential for aerobic alcohol oxidation such as Pd, Rh, Pt, Ru, Ag and Cu were also examined under similar reaction conditions in the deoxygenation of *trans*-stilbene oxide, as shown in Scheme 4. Notably, HT-supported silver nanoparticles also functioned as an efficient catalyst, giving *trans*-stilbene in high yield, while the other metal nanoparticles did not possess any catalytic activity.

The scope of the deoxygenation reaction was then explored with other epoxides, as can be seen in Table 5. Au/HT deoxygenated a wide range of epoxides, affording the corresponding alkenes in excellent yields. For example, epoxides possessing phenyl (entries 1, 8, 10, 15 and 14), alkyl (entries 20, 22, and 25), ether (entry 16), carbonyl (entry 27), hydroxyl (entry 29) and olefinic (entry 31) groups were successfully employed. Interestingly, Ag/HT showed unique chemoselectivity for the deoxygenation; only styrene oxide derivatives proved to be reactive (entries 5, 9, 11, 13 and 15). Furthermore, these solid catalysts were recoverable by simple filtration following the deoxygenation without any loss in their activities or selectivities, even after several reuse experiments (Table 5, entries 2, 3, 6 and 7).

The deoxygenation of epoxides using Au/HT was also efficient on a preparative scale. For example, *trans*-stilbene oxide (3.9 g; 20 mmol) successfully gave *trans*-stilbene (3.4 g; 95% isolated yield) with a turnover frequency (TOF) and a turnover number (TON) of up to 270 h^-1^ and 20,000, respectively (Table 5, entry 4). These values are three orders of magnitude greater than those in previously reported catalyst systems [43,44,45,46,47].

We propose that the deoxygenation proceeds through cooperation between the gold nanoparticles and a basic site of HT (Scheme 5). The first step involves oxidation of the alcohol on Au/HT (I) via abstraction of the proton of the alcohol by a basic site of HT to generate [H-HT]^+^ and a [Au-alcolate] species (II) at the periphery of the AuNP-HT interface. The Au-alcoholate species then undergoes *β*-hydride elimination to give an [H-Au]^–^ species (III) together with the corresponding carbonyl compound. Next, protonation from the [H-HT]^+^ species opens the epoxide, which then is attacked by the [H-Au]^–^ species to give the intermediate (IV). Subsequent dehydration from IV provides the alkene, thereby completing the catalytic cycle.

The above reaction pathway is well supported by the following experimental results: (i) almost equimolar amounts of acetophenone and H_2_O were formed for every mole of styrene during the deoxygenation of styrene oxide using 1-phenylethanol; (ii) when *trans*-2-octenal was used in place of styrene oxide, chemoselective reduction occurred to exclusively afford *trans*-2-octenol, preserving the incipient C=C double bond [48]. It is well-known that all of the metal nanoparticles tested can form metal-hydride species in alcohol oxidation reactions. The distinguished deoxygenation activities of gold and silver nanoparticles from those of other metal NPs can be attributed to their unique reactivity toward an epoxide (Path III→IV→I in Scheme 3). The solid support of HT can promote the deoxygenation as a base (Path I→II in Scheme 3) as well as stabilize these metal nanoparticles.

### 3.2. Deoxygenation of Epoxides Using Au/HT-CO/H_2_O

From an environmental and practical synthetic point of view, the use of water in organic reactions instead of organic solvents is attractive [49,50,51,52,53] because of the low cost, safety (non-explosive, non-flammable and non-toxic), and ease of phase-separation of water-insoluble products in the work-up procedure. For this purpose, our next target focused on the design of a high performance catalyst system for the deoxygenation of epoxides in water.

We found that using CO/H_2_O as a reducing agent along with the Au/HT catalyst provides highly efficient deoxygenation of many epoxides into the corresponding alkenes. The reaction takes place under an atmosphere of CO at room temperature in water without any added organic solvents. [54] Au/HT and styrene oxide were added to water and the heterogeneous mixture was stirred under an atmosphere of CO at room temperature. The reduction of styrene oxide smoothly occurred to give styrene in over 99% yield, accompanied by the formation of an equimolar amount of CO_2_ (Table 6, entry 1). Notably, water was found to be the best solvent to promote efficient deoxygenation, with lower yields being obtained in organic solvents (Table 6 entries 1 vs. 4-6). When Au/HT was removed from the reaction mixture at 50% styrene conversion, continued stirring of the filtrate under similar conditions did not yield any further products. In addition, no gold ions were detected in the filtrate using ICP-AES analysis, confirming the occurrence of the deoxygenation at the active site of the gold nanoparticles on the Au/HT catalyst.

After completing the deoxygenation of styrene oxide, the reaction mixture was separated into the product phase and the aqueous phase containing the hydrophilic solid Au/HT. Styrene was then easily isolated by extraction using n-hexane. The recovered aqueous phase containing Au/HT was reusable without any loss of activity or selectivity during the recycling experiments (Table 6, entries 2 and 3). The substrate scope of Au/HT for the deoxygenation of epoxides is exemplified in Table 6 entries 7-12. Various epoxides were selectively deoxygenated into the corresponding alkenes at room temperature in excellent yields without the use of organic solvents. Notably, *trans*-2,3-epoxy-3-phenyl-1-propanol selectively gave cinnamyl alcohol in excellent yield while maintaining the hydroxyl group, and the selectivity for cinnamyl alcohol is much higher in water than in organic solvents (entry 10 vs. entries 11 and 12). These phenomena are in sharp contrast with the results from our previously reported system of gold-catalyzed deoxygenation using alcohols [42], where the hydroxyl group was not tolerated and cinnamaldehyde was the main product. We believe that a gold-hydride species generated in situ from the reaction of H_2_O with CO is the active species for the deoxygenation of epoxides. [48] The use of D_2_O in place of H_2_O under identical conditions significantly affected the reaction rate for the deoxygenation of styrene; a *k_H_/k_D_* value of 3.9 was observed, supporting the theory that not only CO functions as a sole reductant but also water takes part in the deoxygenation.

To verify the above hypothesis, Fourier Transform Infrared (FT-IR) studies of Au/HT were carried out in the presence of CO and H_2_O. When Au/HT was treated with CO and H_2_O vapor at 298 K, a new band attributed to a gold-hydride species appeared at 1,750 cm^-1 ^[55,56], while no peak was observed at 1,750 cm^-1^ when Au/HT was treated with CO. Furthermore, the band attributed to the gold-hydride species gradually disappeared after exposure to styrene oxide vapor.

**Scheme 6:**
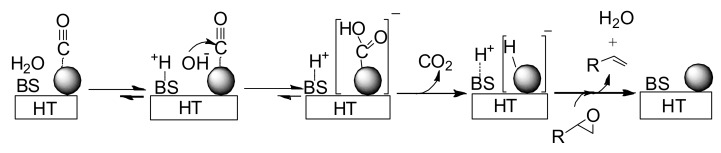
Possible Reaction Pathway for the Au/HT-catalyzed Deoxygenation of an Epoxide in Water Using CO/H_2_O as a Reductant.

Our proposed reaction pathway can be seen in Scheme 6. A basic site on HT surface presumably plays an important role in facilitating formation of the gold-hydride species through nucleophilic attack of OH^-^ on a gold-CO species followed by decarboxylation [57,58,59]. The heterolytic hydrogen species generated on Au/HT then deoxygenates the epoxide to form the corresponding alkene and water.

### 3.3. Deoxygenation of Nitro Compounds Catalyzed by Ag/HT Using CO/H_2_O

We next succeeded in extending our deoxygenation methodology using Olympic medal metal NP catalysts with CO/H_2_O as a reductant to the selective deoxygenation of nitro compounds. Namely, Ag/HT functioned as an effective heterogeneous catalyst for the quantitative chemoselective reduction of various nitroaromatics to the corresponding anilines with >99% selectivity, even in the presence of a C=C double bond. This methodology completely suppresses the reduction of C=C double bonds during the reduction of nitro functionalities [60].

Aniline derivatives are valuable intermediates in the production of agrochemicals, pharmaceuticals and dyes [61]. Although the reduction of aromatic nitro compounds is the most straightforward method for the synthesis of the corresponding anilines [62], it is difficult to reduce only the nitro functionality in the presence of other reducible substituents [63]. The chemoselective reduction of nitro compounds bearing C=C bonds to the corresponding anilines has been achieved using a large excess of stoichiometric reducing agents such as Fe, Sn, Zn and NaS_2_O_4_ [64]. These reaction systems, however, have typically suffered from the production of harmful wastes, the need for neutralization of acid additives used as a hydrogen source and low atom efficiencies. To date, TiO_2_-supported gold NPs (Au/TiO_2_) may be the best heterogeneous catalyst for the chemoselective reduction of the nitro functionality under an H_2_ atmosphere [65].

**Scheme 7:**
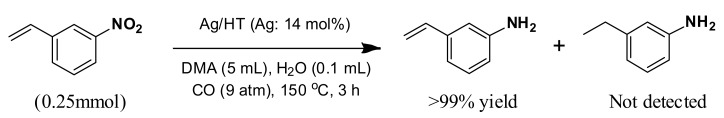
Deoxygenation of 3-Nitrostyrene by Ag/HT Using CO/H_2_O.

With our system, 3-nitrostyrene and Ag/HT were mixed in DMA solvent under 9 atm of CO at 150 °C in the presence of water. 3-Aminostyrene was formed in over 99% yield in 3 h without production of any reduction products of the C=C double bond such as 3-ethylaniline or 3-ethylnitrobenzene (Scheme 7).

**Figure 1:**
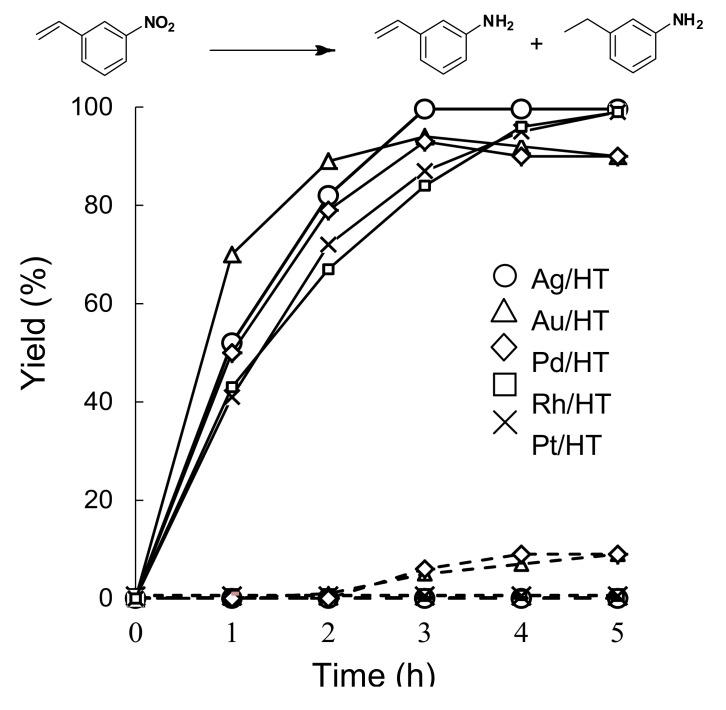
Kinetic Plots Showing the Yield of 3-Aminostyrene (solid lines) and 3-Ethylaniline (dashed lines) for the Reduction of 3-Nitrostyrene using HT-supported Metal Nanoparticles: Ag/HT, Au/HT, Pd/HT, Rh/HT and Pt/HT.

The time courses for the reduction of 3-nitrostyrene using Ag/HT and other HT-supported metal nanoparticles (Au/HT, Pd/HT, Pt/HT and Rh/HT) are shown in Figure 1.

**Scheme 8:**
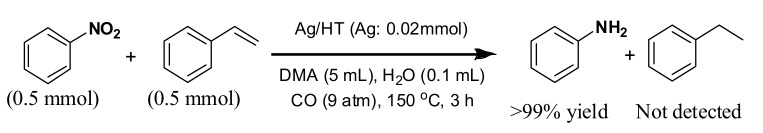
Intermolecular Competitive Reaction of Nitrobenzene and Styrene Using Ag/HT in the Presence of CO/H_2_O.

Although the activity of the Au/HT catalyst was higher than that of Ag/HT, an over reduction to 3-ethylaniline was observed, resulting in lower selectivity toward 3-aminostyrene. Pt/HT and Rh/HT functioned as good catalysts, giving 3-aminostyrene in moderate yields with high selectivity, while Pd/HT did not exhibit high activity or chemoselectivity. These results indicate that Ag/HT gives the highest yield of 3-aminostyrene. Notably, the C=C bond of the product 3-aminostyrene was maintained intact even after the complete conversion of 3-nitrostyrene. This chemoselectivity of Ag/HT towards 3-aminostyrene in the reduction of 3-nitrostyrene is greater than that of previously reported catalyst systems [66,67,68,69,70,71,72]. The high chemoselectivity of Ag/HT for nitro functionalities was further investigated in the intermolecular competitive reaction of nitrobenzene and styrene (Scheme 8).

Interestingly, nitrobenzene was reduced to give aniline in over 99% yield, but styrene was not reduced at all. These results clearly demonstrate that the Ag/HT catalyst system using CO/H_2_O shows complete chemoselectivity for nitro functionalities in the presence of inter- and intra-molecular olefinic functionalities.

This result is in sharp contrast with that obtained for a previously reported Au/TiO_2_ catalyst system in which reduction of styrene did occur in the competitive reduction of nitrobenzene and styrene [69]. Furthermore, the Ag/HT catalyst was applicable to the reduction of various nitro compounds bearing C=C double bonds, such as 4-nitrostyrene, 4-nitrostilbene, 1-nitro-4-propenyl benzene and 5-nitro-indole; the corresponding anilines were obtained in high yields with over 99% selectivity (Table 7).

In separate experiments under identical conditions without 3-nitrostyrene, H_2_ was not generated from the water-gas shift reaction. Moreover, when H_2_ was used as a reducing agent instead of CO/H_2_O, selective reduction of 3-nitrostyrene to 3-aminostyrene did not occur. These results rule out the participation of H_2_ in the Ag/HT-catalyzed reduction reaction described above. [73,74] In-situ silver hydride species generated from the reaction of H_2_O with CO at the surface of the Ag nanoparticles may be the active species that leads to highly chemoselective reduction of nitroaromatics with suppression of the reduction of the olefinic bond.

## 4. Conclusions 

The cooperative effect between Olympic medal metal nanoparticles of gold, silver and copper and the surface base properties of hydrotalcite yields unique catalytic properties for various organic transformations such as oxidation of alcohols, lactonization of diols and deoxygenation of epoxides and nitro aromatics. The hydrotalcite-supported Olympic medal metal nanoparticles offer significant benefits in achieving “green” organic synthesis due to: (i) their high catalytic activities and selectivities; (ii) wide applicability; and (iii) reusability of the catalyst. Integrated heterogeneous catalysts that rely on the concerted action between Olympic medal metal nanoparticles and the surface properties of inorganic crystallites will open new avenues for development of novel organic transformations and contribute to the realization of Green Sustainable Chemistry.

## Figures and Tables

**Figure 2:**
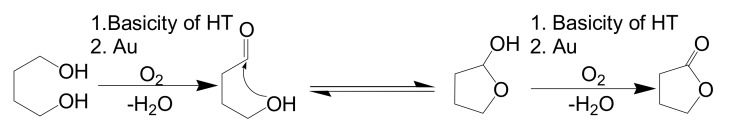
Lactonization Reaction Path.

**Figure 5:**
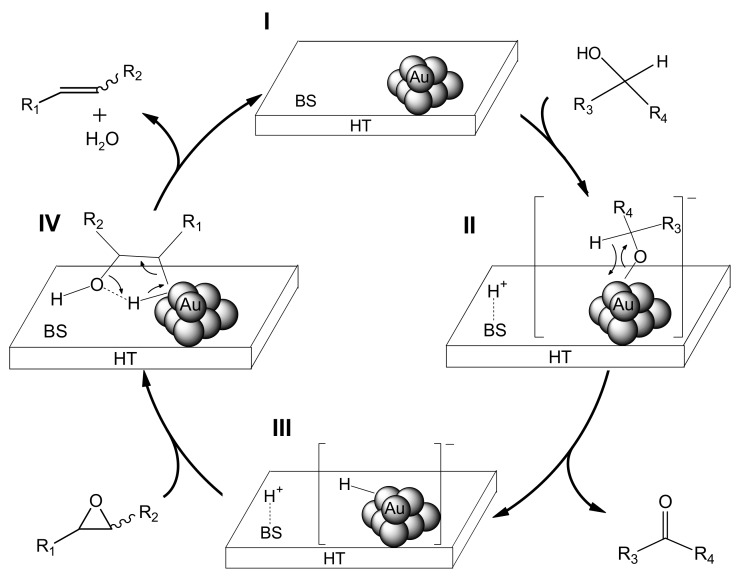
Plausible Reaction Path for the Au/HT-catalyzed Deoxygenation of an Epoxide through the Concerted Action between Au NPs and a Basic Site of HT (represented by BS).

**Table 1:** Oxidation of Various Alcohols by Au/HT^[a]^. 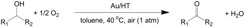

Entry	Substrate	Product	Time (h)	Conv.^[b]^ (%)	Yield^[b]^ (%)
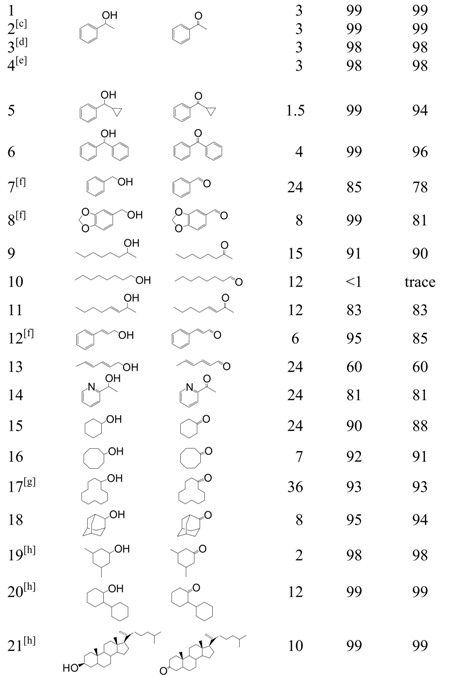					

^[a]^ Reaction conditions: Au/HT(0.1 g, Au: 0.45 mol%), alcohol (1 mmol), toluene (5 mL). ^[b]^ Determined by GC or HPLC using internal standard technique. ^[c]^ Reuse 1. ^[d]^ Reuse 2. ^[e]^ Reuse 3. ^[f]^ The ester was formed as a byproduct. ^[g]^ Alcohol (0.5 mmol). ^[h]^ 80 °C.

**Table 2:** Size-effect of Gold Nanoparticles on the Oxidation of 1-Phenylethanol^[a]^. 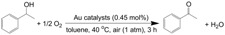

Entry	Substrate	Reducing reagent	Conv.^[b]^ (%)	Yield^[b]^ (%)	Particle Size (nm)
1	Au/HT	KBH4	99	99	2.7
2	Au/HT	H2	99	99	2.7
3	Au/HT	hydrazine	71	69	4.6
4	Au/MgO	KBH4	71	71	3.1
5	Au/MgO	H2	37	37	4.4
6	Au/MgO	hydrazine	18	18	5.7
7	Au/Al2O3	KBH4	71	71	3.6
8	Au/Al2O3	H2	32	32	4.2
9	Au/Al2O3	hydrazine	22	22	5.8
10	Au/TiO2	KBH4	16	14	3.7
11^[c]^	Au/TiO2 + Na2CO3	KBH4	65	65	3.7
12	Au/SiO2	KBH4	<1	<1	14

^[a]^ Reaction conditions: Au catalyst (0.45 mol%), 1-phenylethanol (1 mmol), toluene (5 mL). ^[b]^ Determined by GC using internal standard technique. ^[c]^ Na_2_CO_3_ (3 mmol) was added.

**Table 3:** Oxidation of Various α, ω-Diols Using Au/HT^[a]^. 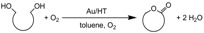

Entry	Substrate	Product	Temp. (°C)	Time (h)	Conv.^[b]^ (%)	Yield^[b]^ (%)
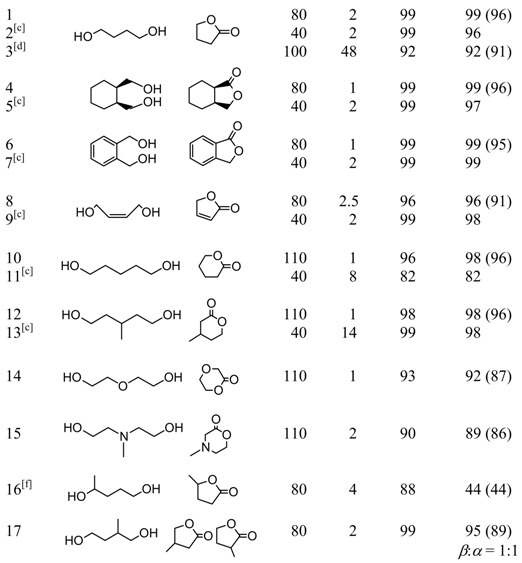						

^[a]^ Reaction conditions: Au/HT (0.1 g, Au: 0.45 mol%), diol (1 mmol), toluene (5 mL). ^[b]^ Determined by GC and LC using an internal standard technique; values in parentheses are the yields of the isolated products. ^[c]^ Substrate (0.5 mmol). ^[d]^ Substrate (70 mmol), Au/HT (1.0 g, Au: 0.06 mol%), toluene (180 mL), DMA (5 mL). ^[e]^ Substrate (0.3 mmol). ^[f]^ 5-Hydroxy-2-pentanone and 4-oxo-pentanal were formed as byproducts.

**Table 4:** Dehydrogenation of Alcohols Using Cu/HT^[a]^.

Entry	Substrate	Product	Temp.(°C)	Time (h)	Conv.^[b]^ (%)	Yield^[b]^ (%)
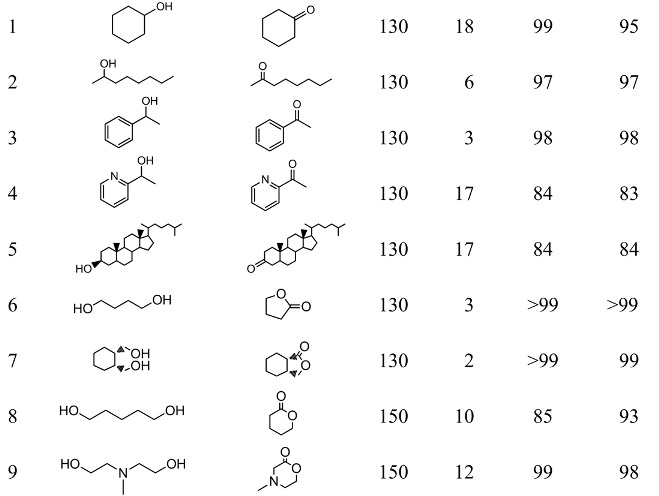						

^[^^a^^]^ Reaction conditions: Cu/HT (0.10 g, Cu: 0.071 mmol), substrate (1.0 mmol), mesitylene (5.0 mL). ^[^^b^^]^ Determined by GC using an internal standard technique.

**Table 5:** Deoxygenation of Various Epoxides Using Au/HT and Ag/HT^[a]^.

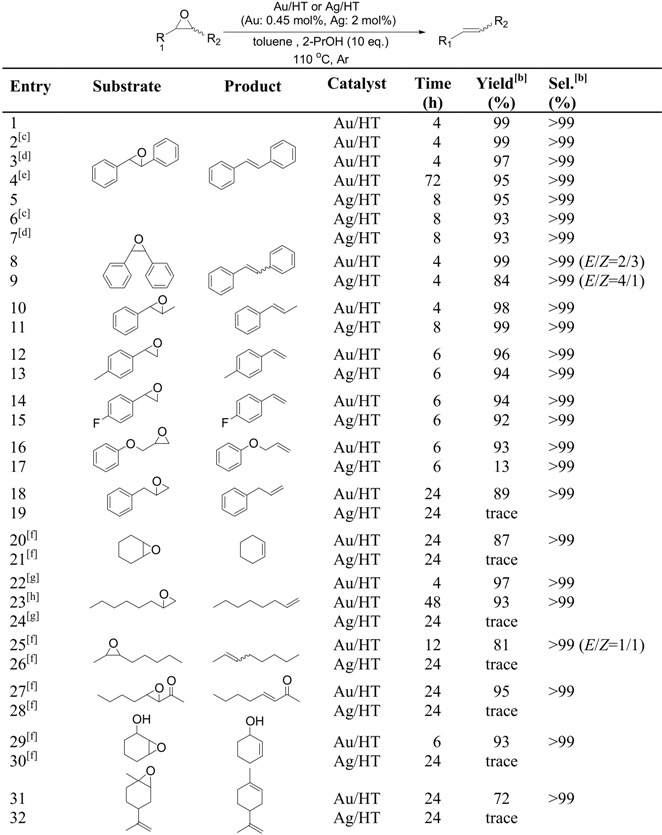

^[^^a^^]^ Reaction conditions: Catalyst (0.1 g), substrate (1 mmol), toluene (5 mL), 2-propanol (0.6 mL). ^[^^b^^]^ Determined by GC and LC using internal standard technique. ^[^^c^^]^ Reuse 1. ^[^^d^^]^ Reuse 2. ^[^^e^^]^ Catalyst (0.02 g), substrate (20 mmol), 2-propanol (20 mL), 150 °C. ^[^^f^^]^ Substrate (0.3 mmol), catalyst (0.2 g). ^[^^g^^]^ 80 °C. ^[^^h^^]^ Catalyst (0.02 g), substrate (10 mmol), 2-propanol (20 mL).

**Table 6:** Deoxygenation of Various Epoxides in Water Using the Au/HT Catalyst ^[a]^.

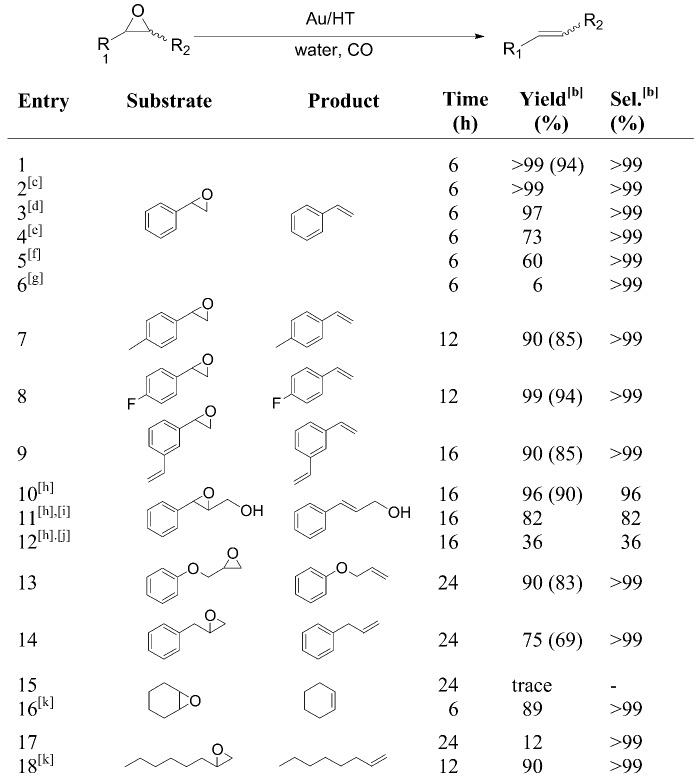

^[a] ^Reaction conditions: Substrate (0.5 mmol), Au/HT (0.1 g, Au: 0.9 mol%), water (5 mL), CO (1 atm), 27 °C. ^[b]^ Determined by GC using internal standard technique. Values in parentheses are the isolated yields. ^[c]^ Reuse 1. ^[d]^ Reuse 2. ^[e]^ Toluene (5 mL), water (0.1 mL). ^[f]^ THF (5 mL), water (0.1 mL). ^[g]^ DMA (5 mL), water (0.1 mL). ^[^^h]^ Cinnamaldehyde was formed as a byproduct. ^[^^i]^ Toluene (5 mL) was used as a solvent. ^[^^j]^ THF (5 mL) was used as a solvent. ^[^^k]^ THF (5 mL), water (0.1 mL), CO (5 atm), 110 °C.

**Table 7:** Deoxygenation of Various Nitro Compounds by Ag/HT Using CO/H_2_O^[a]^. 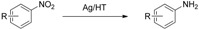

Entry	Substrate	Product	Time (h)	Conv.^[b]^ (%)	Sel.^[b]^ (%)
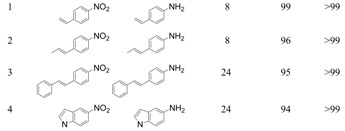

^[a]^ Reaction conditions: Ag/HT (14 mol%), substrate (0.25 mmol), DMA (5 mL), H_2_O (0.1 mL), 150 °C, CO (9 atm). ^[^^b^^]^ Determined by GC using an internal standard technique.
